# *Flowering Date1*, a major photoperiod sensitivity gene in adzuki bean, is a soybean floral repressor *E1* ortholog

**DOI:** 10.1270/jsbbs.21051

**Published:** 2022-02-02

**Authors:** Yusuke Imoto, Shoko Yoshikawa, Yuki Horiuchi, Takumi Iida, Taisei Oka, Shuichi Matsuda, Yoshihiko Tokuji, Masahiko Mori, Kiyoaki Kato

**Affiliations:** 1 Department of Agro-Environmental Science, Obihiro University of Agriculture and Veterinary Medicine, Nishi 2-11 Inada, Obihiro, Hokkaido 080-8555, Japan; 2 Tokachi Agricultural Experiment Station, Agricultural Research Department, Hokkaido Research Organization, Memuro, Hokkaido 082-0081, Japan; 3 Department of Human Sciences, Obihiro University of Agriculture and Veterinary Medicine, Nishi 2-11 Inada, Obihiro, Hokkaido 080-8555, Japan

**Keywords:** adzuki bean, photoperiod response, major gene, *Flowering Date1*, *E1*

## Abstract

Adzuki bean is an important legume crop originating in temperate regions, with photoperiod in sensitivity being a key factor in its latitudinal adaptation. The *Flowering Date1* (*FD1*) gene has a large effect on the photoperiodic response of flowering time, but the molecular basis for the effect of this locus is undetermined. The present study delimited the *FD1* locus to a 17.1 kb sequence, containing a single gene, an *E1* ortholog (*VaE1*). A comparison between *Vigna angularis* ‘Shumari’ (photoperiod insensitive) and ‘Acc2265’ (photoperiod sensitive) identified 29 insertions/deletions and 178 SNPs upstream of *VaE1* in the *FD1* locus. *VaE1* expression in ‘Acc2265’ was greater under long-day than short-day conditions, whereas *VaE1* expression in ‘Shumari’ was lower regardless of day length. These findings suggested that responsible gene of *FD1* is a *VaE1*, which acts as a floral repressor by being upregulated in response to long-day conditions. The inability to upregulate *VaE1* under long-day conditions was linked to its ability to flower under these conditions. These results provide greater understanding of the molecular control of a flowering date and clues enabling the breeding of adzuki bean at higher latitudes.

## Introduction

The adzuki bean, *Vigna angularis* (Willd.) Ohwi and Ohashi, is a traditional legume grown throughout East Asia and northern South Asia (Lumpkin and McClary 1994, Zong *et al.* 2003). In Japan, adzuki beans are the second most important legume after soybeans, with about 90% of adzuki beans grown in Hokkaido prefecture, the northernmost island in Japan (NL45-42°). In Hokkaido, adzuki beans are planted in late May and harvested in mid-September to early October, thus avoiding damage at low temperatures. Flowering begins in late July, when the natural length of the day, including twilight, reaches a maximum of 16 h. Because adzuki bean is primarily a short-day annual species, most adzuki bean cultivars have a short-day requirement for floral induction, with flowering normally delayed under long-day conditions. To flower under long-day conditions, adzuki bean cultivars grown at higher latitudes, including in Hokkaido, must have adapted to long days by becoming insensitive to these conditions.

Soybean [*Glycine max* (L.) Merr.] is an important model short-day legume plant used to assess photoperiodism (Owen 1927). To date, more than ten major loci affecting time of flowering and maturity have been identified in soybean (Samanfar *et al.* 2017). The *E1* gene is thought to have the greatest effect on determination of flowering time under field conditions, delaying plant maturity (Xia *et al.* 2012). Varietal adaptability can therefore be improved by reducing the function of the *E1* gene. *E1* (*Glyma.06g207800*; Gmax275 (ver. 2.0)) is intron-free and encodes a protein containing a putative bipartite nuclear signal (NLS) and a domain distantly related to the plant specific B3 domain (B3-like domain) (Xia *et al.* 2012). *E1* is expressed in a bimodal pattern, with higher expression under long-day (LD) than short-day (SD) conditions. *E1* is a putative transcription factor that negatively controls two florigen genes, *GmFT2a* and *GmFT5a*, to delay flowering under a background that includes with functional *PHYA* genes (encoded by *E3* and *E4*) and LD conditions. *E1* was also shown to positively regulate another florigen gene, *GmFT4*, to control flowering in soybean (Zhai *et al.* 2014), and two *E1* orthologs, *E1La* (*Glyma.04G156400.1*) and *E1Lb* (*Glyma.04G143300.1*), were found to control the onset of flowering (Xu *et al.* 2015). Therefore, light-induced *E1* transcription mediated by PHYA (*E3* and *E4*) may play a central role in the control of photoperiodic responses of flowering in soybean, distinct from pathways in Arabidopsis and rice (Xu *et al.* 2015). Wild *Lotus japonicus* plants from various geographic locations throughout Japan that differ in flowering times have shown a polymorphism, *Lj5g3v2221340*, in the *LjE1L* gene, an ortholog of *E1*, suggesting that natural variations in *LjE1L* are associated with flowering in wild *L. japonicus* (Wakabayashi *et al.* 2014). However, the ability of natural variations of *E1* orthologs to regulate photoperiodic flowering responses in other legumes remains unclear.

Several loci associated with days to first flowering in adzuki bean have been identified under natural day-length conditions (Isemura *et al.* 2007, Kaga *et al.* 2008, Li *et al.* 2017, Liu *et al.* 2016, Yamamoto *et al.* 2016). *Flowering Date1* (*FD1*) is a major gene controlling photoperiod sensitivity (Yamamoto *et al.* 2016). *V. angularis* ‘Shumari’, grown in Hokkaido, carries a photoperiod insensitive *FD1* allele, whereas ‘Acc2265’ carries a photoperiod sensitive *FD1* allele. The *FD1* locus has been mapped to a 2.8 Mb sequence between two molecular markers, Az02-37M3 and Az02-40M9, on chromosome 2 (Yamamoto *et al.* 2016). Based on the adzuki bean genome sequence (Vigna Genome Server, https://viggs.dna.affrc.go.jp/), the *FD1* region is thought to contain a total of 223 genes (Yamamoto *et al.* 2016). Candidates for the *FD1* locus include the sequences, *Vigan.02G254400.01* (similar to TOC1 [H8YHW5 in *Phaseolus vulgaris*]), *Vigan.02G275000.01* (similar to FAR1-RELATED SEQUENCE 4-like protein [XP_006598422.1 in *G. max*]), *Vigan.02G285600.01* (similar to phytochrome [V7AY23 in *P. vulgaris*]) and *Vigan.02G286700.01* (similar to period circadian protein, putative isoform 1 [XP_007049424.1 in *Theobroma cacao*]) (Yamamoto *et al.* 2016). However, the molecular basis for the *FD1* locus is undetermined. The present study utilized map-based cloning to clarify the molecular basis of the *FD1* locus. The *FD1* locus was delimited to a 17.1 kb region containing a single gene, *E1* ortholog (*VaE1*). Gene expression analysis showed that the expression of *VaE1* was negatively correlated with flowering date, acting as a floral repressor in response to longer day-length. The *fd1* allele, which is associated with constitutively lower expression of *VaE1* independent of photoperiod, was shown to be involved in the gain of photoperiod insensitivity in adzuki bean.

## Materials and Methods

### Mapping population

The adzuki bean [*Vigna angularis* (Willd.)] plants used in this study included the cultivar ‘Shumari’, grown in Hokkaido, and a landrace ‘Acc2265’, a long-day sensitive genotype (Aoyama and Shimada 2014, Yamamoto *et al.* 2016). For genetic mapping, a total of 3,799 F_2_ plants of a cross between ‘Shumari’ and ‘Acc2265’ and self-pollinated F_3_ progeny were used.

### Growth conditions

For the initial mapping, 1,525 F_2_ and ‘Shumari’ plants were planted in the experimental field at the Tokachi Agricultural Experiment Station (TAES; 42°91ʹN, 143°05ʹE), Hokkaido Research Organization, on 24 May 2016. Because ‘Acc2265’ did not reach flowering at the end of September under natural day length at Obihiro University of Agriculture and Veterinary Medicine (OUAVM; 42°52ʹN, 143°9ʹE) (Yamamoto *et al.* 2016), the flowering date of ‘Acc2265’ was not evaluated in the present field trial. Plants were monitored every 2 or 3 days to determine their first flowering date. Young leaves from F_2_ plants were collected to isolate DNA. For F_3_ progeny test, eight individuals derived from each F_2_ plant were planted in a 2.0 L plastic pot and grown in the glass house at OUAVM from the end of May to August 2017 under natural day length. Plants were monitored every day to determine their first flowering date.

For fine mapping, 2,274 F_2_ plants were planted four times during 2020 in every other cell of a cell plug tray (cell count, 8 × 16; tray size, 52 cm × 25 cm; cell size, 3 cm × 3 cm; cell depth, 4.4 cm) filled with soil compost. Specifically, 322 F_2_ plants were planted on 26 May, 824 on 8 June, 127 on 29 June and 1,001 on 8 July in the glass house at OUAVM and grown under natural day length. Young leaves of each plant were collected to isolate DNA and the F_2_ plants carrying recombinant F_2_ plants between the two target DNA markers flanking *FD1* were screened. Selected F_2_ plants were transplanted to 2.0 L plastic pots and their first flowering date determined. For progeny tests to determine *FD1* genotypes, 9-70 F_3_ plants of each of 70 F_2:3_ lines were planted on 18 November 2020 in every other cell of a cell plug tray (cell count, 8 × 16; tray size, 52 cm × 25 cm; cell size, 3 cm × 3 cm; cell depth, 4.4 cm) filled with soil compost. Plants were grown under long day (>20 hr) conditions, consisting of a combination of natural day length and additional illumination with fluorescent light in the greenhouse at OUAVM, with temperature maintained over 15°C. Plants were monitored every day to determine their first flowering date.

### InDel and SNP marker analysis

Insertion-deletion (InDel) and single nucleotide polymorphism (SNP) markers were developed by whole genome resequencing. The genome of ‘Acc2265’ was sequenced with the Illumina HiSeq X Ten (Illumina, San Diego, CA, USA) by Novogene (Beijing, China). All the clean reads were mapped to the ‘Shumari’ genome sequence (https://viggs.dna.affrc.go.jp) using BWA (version 0.7.8) software (Li and Durbin 2009) with default parameters. The average depth of mapped reads at each site was 39 X. The InDel and SNP sites were detected using SAMtools (version 0.1.19) software (Li *et al.* 2009). Only fragments with an insertion or deletion more than 4 bp in size were used to design InDel markers. Primers 20–27 nucleotides in length (optimal length, 22 nucleotides), with melting temperatures (Tm) of 58–61°C (optimal Tm, 60°C) and yielding products 100–400 bps in length, were designed using Primer 3.0 (v. 0.4.0) software (Untergasser *et al.* 2012, https://bioinfo.ut.ee/primer3-0.4.0/) (Supplemental Table 1). For SNP genotyping, SNPs were converted to cleaved amplified polymorphic sequence (CAPS) markers and derived cleaved amplified polymorphic sequence (dCAPS) markers using the web-based free software program dCAPS Finder 2.0 (Neff *et al.* 2002) to identify appropriate restriction enzymes to detect each SNP. Appropriate PCR primer sets flanking each target SNP were designed using Primer 3.0 (v. 0.4.0) software (Supplemental Table 1).

Young leaves of parents, the mapping population and accessions were collected, and their DNA was extracted and subjected to marker analyses as described (Yamamoto *et al.* 2016).

### Survey of the *FD1* allele in Japanese and Chinese accessions

The *FD1* genotype was surveyed in 104 accessions, including landraces, cultivars, breeding lines and weedy adzuki beans in Japan, and in ‘Shumari’ and ‘Acc2265’ (Supplemental Table 2). In addition, the *FD1* genotype was surveyed in 23 accessions representing landraces in China.

To test associations between the *FD1* locus and photoperiod, days to flowering under SD and LD conditions were evaluated in 73 accessions. Seeds were planted in 300 mL plastic pots filled with soil compost and incubated at 25°C for 4 days in the dark with adequate watering in a growth cabinet. Seedlings were separated into two groups, with three individuals each grown at 25°C under SD (8 h of light, 6:00 to 14:00/16 h of darkness, 14:00 to 6:00) and LD (16 h of light, 6:00 to 22:00/8 h of darkness, 22:00 to 6:00) conditions. Days to first flowering after sowing (DAS) were determined. To calculate the average DAS of each set of three individuals, individuals without flowers at 60 DAS were assigned a flowering date of 61 DAS.

### RNA extraction and reverse transcription quantitative PCR (RT-qPCR)

Seeds were planted in 500 mL plastic pots filled with 420 mL soil compost and incubated at 25°C for 4 days in the dark with adequate watering in a growth cabinet. Seedlings were separated into two groups, one grown under SD conditions (8 h of light, 6:00 to 14:00 /16 h of darkness, 14:00 to 6:00) and the other under LD conditions (16 h of light, 6:00 to 22:00/8 h of darkness, 22:00 to 6:00). Plants grown under each condition were randomly arranged every day to avoid positional effects. Pieces of fully developed trifoliate leaves at the top of plants were sampled and bulked from three plants for each biological sample from 8:00 to 10:00 on 30 days after planting, because the exact zeitgeber time (after dawn) for sampling was considered the first peak of a bimodal diurnal pattern for soybean *E1* expression appearing around 2 to 4 hr after dawn (Xia *et al.* 2012, Xu *et al.* 2015). Five biological samples were prepared. Sampled tissues were immediately frozen in liquid N_2_ and stored at –80°C.

Total RNA was isolated from frozen tissues using TRIzol RNA Isolation Reagents (Thermo Fisher Scientific), according to the manufacturer’s instructions. cDNA was synthesized using PrimeScript^TM^ II 1st strand cDNA Synthesis Kit (Takara Bio) and oligo dT primers. Each qPCR mixture contained 2.0 μL of cDNA, 0.8 μL of 10 μM mixed primers, 10 μL SYBR Premix Ex Taq Ⅱ (Takara Bio), and water to a final volume of 20 μL. Quantitative PCR was performed using the 7300 Real-Time PCR System (Applied Biosystems). The primers for VaE1 consisted of 5ʹ-CATCTCCCCCAAAATCCTC-3ʹ (forward) and 5ʹ-TCGCTTTAGGACGAGTTGGT-3ʹ (reverse), and the amplification protocol consisted of 40 cycles of denaturation at 95°C for 5 s and annealing and extension at 68°C for 30 s. As an internal control, the level of ubiquitin mRNA (Vigan.07G221100.01, Chi *et al.* 2016) was assessed by RT-qPCR using the primers, 5ʹ-CCGGATCAAGGAACGTGTAG-3ʹ (forward) and 5ʹ-AGCAAGCTGCTTACCTGCAT-3ʹ (reverse). A reaction mixture without reverse transcriptase was included to confirm the absence of genomic DNA contamination. Amplification of a single target DNA species was confirmed by dissociation curve analysis of qPCR and gel electrophoresis of the PCR products.

### Sequence alignment and phylogenetic analysis

Protein sequences similar to Vigan.02G276800.01 were identified by searching with NCBI-BLAST (https://www.ncbi.nlm.nih.gov), Phytozome v10.3 (https://phytozome-next.jgi.doe.gov) and the Legume Information System (LIS, https://legumeinfo.org). Conserved protein sequences of the B3-like domain for phylogenic analysis were searched using the online NCBI conserved domain database (https://www.ncbi.nlm.nih.gov/Structure/cdd/cdd.shtml). Multiple sequence alignments were performed using the Clustal W program with default parameters in MEGA v10.1 with some manual editing. An unrooted neighbor-joining phylogenic tree was constructed with 1,000 bootstrap repetitions.

## Results

### Photoperiod insensitivity is controlled by a recessive allele at *FD1* locus

In the 2016 trial, flowering of F_2_ individuals started on 27 July, similar to that of ‘Shumari’ at 29 July (Supplemental Table 3), with 349 F_2_ individuals developing first flowers until 12 August. More than half of these 349 F_2_ individuals developed first flowers on 1 August, with two plants developing first flowers on 12 August, providing further evidence for discontinuous distribution (Yamamoto *et al.* 2016). The F_3_ progeny test representing all 349 F_2:3_ lines found that 349 were fixed as early flowering type. Thus, late and early flowering individuals fit the 3:1 Mendelian segregation ratio (χ^2^_(3:1)_ = 2.92, *p* = 0.09). These results indicated that the early flowering (photoperiod insensitive) phenotype is controlled by a recessive *FD1* allele (*fd1*).

### Fine mapping of the *FD1* locus

**Fig. 1.**
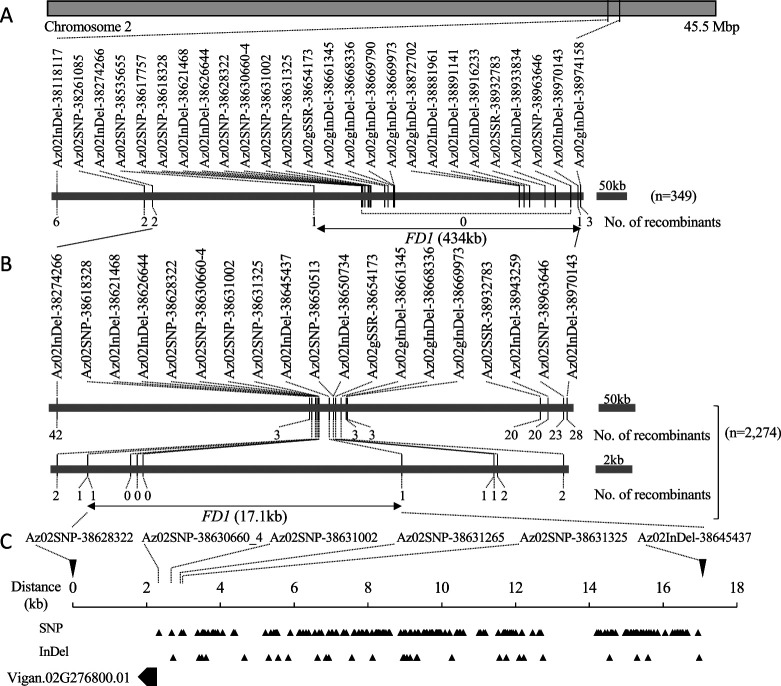
Map-based cloning and characterization of the *FD1* locus. (A) Initial genetic map of *FD1* using 349 *fd1fd1* F_2:3_ lines. (B) Fine scale map of *FD1* using 70 recombinant F_2:3_ lines between Az02InDel-38274266 and Az02InDel-38645437 from 2274 F_2_ individuals. (C) Physical map of Vigan.02G276800.01, representing a single intron-free gene within the delimited region in ‘Shumari’. *Triangles* represent SNPs and InDels of ‘Acc2265’ compared with ‘Shumari’.

Using 349 *fd1fd1* F_2:3_ lines, we initially mapped *FD1* to a 434 kb interval between two molecular markers, Az02SNP-38535655 and Az02InDel-38970143, on chromosome 2 (Fig. 1A). To narrow the candidate region of *FD1*, 2,274 F_2_ individuals were screened for recombinants between two agarose-resolvable Indel markers, Az02gInDel-38274266 and Az02InDel-38970143. Seventy recombinant F_2_ individuals were selected and their genotypes at *FD1* were evaluated based on the segregation patterns of initial flowering date in the F_3_ progeny. In addition, 17 DNA markers were genotyped in these 70 F_2_ individuals (Supplemental Fig. 1). *FD1* was eventually mapped to the region between Az02SNP-38628322 and Az02gInDel-38645437, corresponding to a physical distance of 17.1 kb (Fig. 1B).

Based on the ‘Shumari’ reference genome sequence (Sakai *et al.* 2016, Vigna Genome Server, https://viggs.dna.affrc.go.jp), Az02SNP-38628322 was located 1,806 bp downstream from *Vigan.02G276800.01* and Az02gInDel-38645437 was located 14,785 bp upstream from the transcription start site of *Vigan.02G276800.01*. Thus, the region delimited by fine mapping contained only *Vigan.02G276800.01* (Fig. 1C). Based on the whole genome resequencing data of ‘Acc2265’, a total of 178 SNPs and 29 InDels were detected in the *FD1* locus (Fig. 1C). All polymorphic sequences in the *FD1* locus located upstream of Vigan.02G276800.01. The sequences of the coding region of ‘Acc2265’ were identical to those of ‘Shumari’.

### Phylogenetic analysis of Vigan.02G276800.01 and its homologs

In the Vigna Genome Server (https://viggs.dna.affrc.go.jp), Vigan.02G276800.01 is predicted to be an intron-free gene encoding a protein of 174 amino acids, annotated as “Similar to Uncharacterized protein. [K7MS71, *G. max*]”. BLASTP searches using the 174 amino acid sequence of Vigan.02G276800.0 as a query indicated that the amino acid sequence of Vigan.02G276800.01 is 92% identical to the E1 protein of *G. max* (Xia *et al.* 2012).

**Fig. 2.**
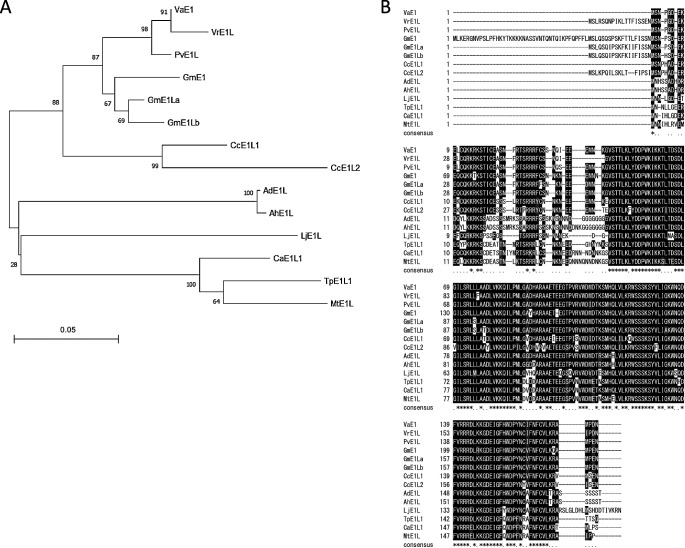
Phylogenetic relationship and sequence alignment of E1-like proteins. Amino acid sequences of *Phvul.009G204600* (*PvE1L*) from common bean, *C. cajan_45915* (*CcE1L1*) and *C. cajan_26468* (*CcE1L2*) from pigeon pea, *Ca_21849* (*CaE1L*) from chickpea, *Medtr2g058520* (*MtE1L*) from *M. truncatula*, *LjE1L* from *L. japonicus*, *LOC112784564* (*AhE1L*) from peanut (*Arachis hypogaea*), *LOC107470881* (*AdE1L*) from *A. duranensis*, *L195_g038525* from red clover (*Trifolium pratense*), *Vigan.02G276800.01* (*VaE1*) from adzuki bean, *Vradi02g13810.1* (*VrE1L*) from *V. radiata*, and *E1*, *E1La* and *E1Lb* from soybean were compared. (A) Phylogenetic tree of *E1*-like genes. The full-length amino acid sequences of E1-like proteins were aligned using Clustal W and the phylogenetic tree was constructed using the neighbor-joining method in MEGA 10.1 (Bootstrap = 1,000). Two main groups were identified, Group I, corresponding to genes from the Millettioid/Phaseoloid clade, and Group II, corresponding to genes from the Hologalegina clade. (B) Amino acid sequence alignment of ten *E1*-like genes from legumes. The B3-like domain is underlined. Putative bipartite nuclear localization signals (NLS) are shown in dotted parentheses, and putative helices are shown as arrows (Zhang *et al.* 2016).

The phylogram of the Legume Information System (LIS) showed that there are 14 *E1*-like genes (amino acid identity >70% to E1) in legumes, *Phvul.009G204600* (*PvE1L*) from common bean, *C. cajan_45915* (*CcE1L1*) and *C. cajan_26468* (*CcE1L2*) from pigeon pea, *Ca_21849* (*CaE1L*) from chickpea, *Medtr2g058520* (*MtE1L*) from *Medicago truncatula*, *LjE1L* from *L. japonicus*, *LOC112784564* (*AhE1L*) from peanut (*Arachis hypogaea*), *LOC107470881* (*AdE1L*) from *Arachis duranensis*, *L195_g038525* from red clover (*Trifolium pratense*), *Vigan.02G276800.01* (*VaE1*) from adzuki bean, *Vradi02g13810.1* (*VrE1L*) from *V. radiata*, and *E1*, *E1La* and *E1Lb* from soybean (Zhang *et al.* 2016). Phylogenetic comparisons of these E1-like proteins showed that these 14 proteins could be divided into two main groups, with Group I consisting of VaE1 (FD1, Vigan.02G276800.01), VrE1L, E1, E1La, E1Lb, PvE1L, CcE1L1 and CcE1L2, and Group II of AdE1L, AhE1L, LjE1L, MtE1L, TpE1L and CaE1L (Fig. 2A). Most *E1*-like genes were predicted to contain no introns, but to possess a putative bipartite NLS near the N-terminus; this bipartite NLS was found to be composed of the KKRK and RRR basic domains at either end separated by 12 amino acid residues (Fig. 2B). Moreover, all genes were predicted to contain a B3-like domain (Fig. 2B). The alignment showed that most residues were highly conserved except for the short extensions at the N- and C-termini.

### Expression analysis of *VaE1*

**Fig. 3.**
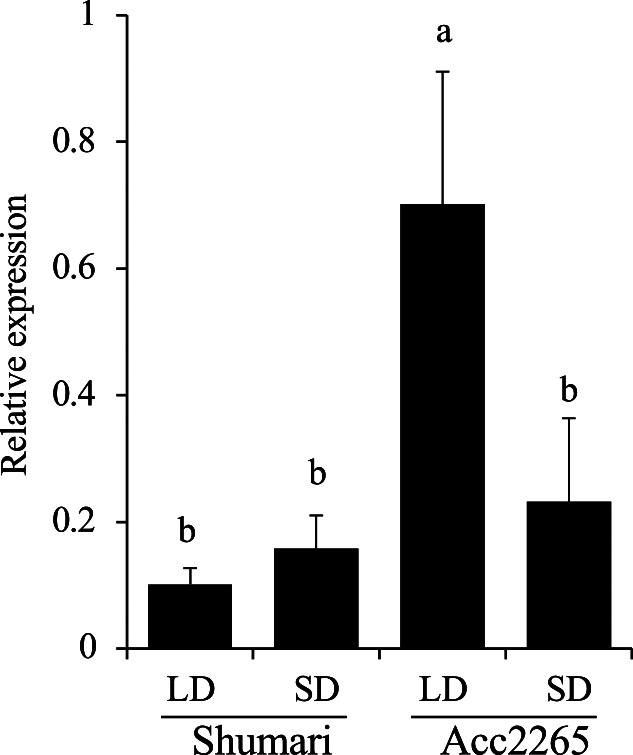
Quantitative reverse transcription PCR analysis of *Vigan.02G276800.01* (*VaE1*) expression levels in ‘Shumari’ and ‘Acc2265’ under long day (LD; 16h light/8h dark) and short day (SD; 8 light/16 dark) conditions. Relative mRNA levels are expressed as ratios to levels of ubiquitin mRNA (Vigan.07G221100.01, Chi *et al.* 2016). *Error bars* represent the standard deviation of the mean of five biological replicates (independent plants). Different letters above the bars represent significant differences among samples (Tukey-Kramer test, *p* < 0.05).

To determine whether *VaE1* is associated with flowering time in response to day-length, the amounts of *VaE1* expressed under long-day (LD) and short-day (SD) conditions in ‘Shumari’ and ‘Acc2265’ were evaluated (Fig. 3). *VaE1* was expressed at a low level in ‘Shumari’, regardless of day length, whereas its expression in ‘Acc2265’ was much higher under LD than SD conditions, suggesting that higher expression of *VaE1* was associated with late flowering of ‘Acc2265’ under LD conditions. These findings suggested that *FD1* is *VaE1* (an *E1* ortholog) which is upregulated by LD conditions and acts as a floral repressor in ‘Acc2265’. In contrast, the inability to upregulate *VaE1* under LD conditions was associated with the ability of ‘Shumari’ to flower under these conditions.

### Cis-element analysis

Cis regulatory elements play essential roles in regulating gene expression. In eukaryotes, temporal and spatial gene expression is governed by the binding of transcription factors to cis-elements (Priest *et al.* 2009). The distributions of light- and circadian clock-related cis-elements in 3.0 kb promoters upstream of Vigan.02G276800.01 were compared in ‘Shumari’ (*fd1*) and ‘Acc2265’ (*FD1*) using NEW PLACE webserver (Higo *et al.* 1999). Seven main cis-elements were identified, including -10PE, CCAAT, E-Box, GATA, GT1, I-Box and INR (Table 1). Of these seven motifs, CCAAT and E-Box are thought to play important roles in regulating the flowering process (Ben-Naim *et al.* 2006, Wenkel *et al.* 2006). Thirteen CCAAT motifs were found in ‘Shumari’ and 12 in ‘Acc2265’, with 22 E-box motifs were found in each genotype. In addition, ‘Shumari’ also harbors five -10PE and 11 INR motifs. Whereas ‘Acc2265’ harbors four -10PE and 12 INR motifs. The difference in cis-elements may be responsible for the differences between the alleles in the levels of expression of Vigan.02G276800.01.

### Survey of the genotype at the *FD1* locus in adzuki bean accessions

**Fig. 4.**
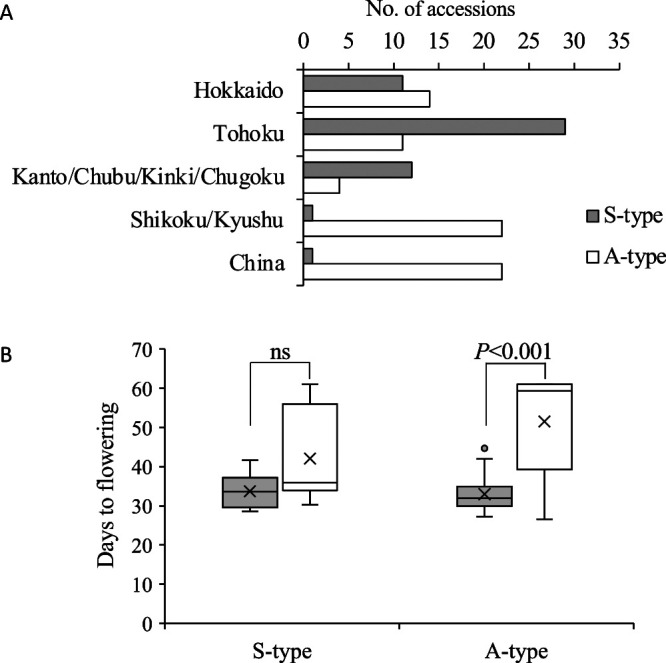
Distributions of genotype frequencies at the *FD1* locus and association between FD1 genotypes and photoperiodism. (A) Regional frequency distributions of the *FD1* genotype. The *FD1* genotype of each accession was estimated using Az02SNP-38631265 and Az02InDel-38627816. S-type and A-type represent ‘Shumari’ type and ‘Acc2265’ type, respectively. (B) Quartile box plots showing days to flowering of S-type (n = 21) and A-type (n = 52) accessions under short day (SD; 8 light/16 dark, gray colored box-plot) and long day (LD; 16h light/8h dark, white colored box-plot) conditions at a temperature of 25°C. The interquartile regions, medians, averages, ranges and outliers are indicated by the boxes, horizontal lines, crosses, vertical lines, and circles, respectively.

To determine whether the *FD1* alleles are region specific, genotypes were surveyed in 129 accessions, 55 of ‘Shumari’ (S-type) and 74 of ‘Acc2265’ (A-type), using the dCAPS marker, Az02SNP-38631265, located 613-bp upstream from the transcription start site (38,631,265-bp position on chromosome 2), and the InDel marker, Az02InDel-38627816, located 2,312 bp downstream of the 3ʹ end of the coding region (38,627,816-bp position on chromosome 2). No recombination between the two molecular markers was observed in any of the 129 accessions (Supplemental Table 2). Of the 105 Japanese germplasms, 54 accessions were S-type and 51 were A-type. S-type accessions were predominant in the Kanto/Chubu/Kinki/Chugoku (75%) and Tohoku (72.5%) regions (Fig. 4A), as well as constituting 44.8% of the accessions in the Hokkaido region (prefecture), but only 4.3% in the Kyushu/Shikoku regions and in accessions from China.

To determine whether the *FD1* allele is associated with flowering time in response to day-length, flowering date were evaluated in 73 accessions under SD (8 hr day-length) and LD (16 hr day-length) conditions (Supplemental Table 2, Fig. 4B). Both genotypes exhibited a narrower range of the days to flowering under SD conditions, although an outlier existed. Under LD conditions, days to flowering were diverse in both genotypes. A-type accessions (n = 52) flowered later than S-type accessions (n = 21) under LD conditions (*p* < 0.01). Furthermore, the average number of days to flowering of S-type accessions did not differ significantly under SD and LD conditions, although some accessions were photoperiod sensitive. The average of days to flowering of A-type accessions was significantly later under LD than under SD conditions (*p* < 0.001), although some accessions were photoperiod insensitive. These observations indicate the genotype at the *FD1* locus is involved, at least in part, in the variations of photoperiod sensitivity of adzuki beans.

## Discussion

The present study demonstrated that the major photoperiod response gene in adzuki bean, *FD1*, is an *E1* ortholog, named *VaE1*. The soybean genome contains three genes of the *E1*-like, *E1*, *E1La* and *E1Lb*, on chromosomes 6 and 4 (Xia *et al.* 2012, Xu *et al.* 2015). These copies were derived from two independent duplication events. The first duplication event gave rise to *E1* and the ancestor of *E1La* and *E1Lb*, whereas the second segmental duplication event led to the generation of *E1La* and *E1Lb* on chromosome 4 (Zhang *et al.* 2016). Ortholog search based on the draft sequencing data of the entire adzuki bean genome identified a total of 1,501 duplicated syntenic blocks (Yang *et al.* 2015). However, there are no other sequences similar to *FD1* in adzuki bean genome (Vigna Genome Server, https://viggs.dna.affrc.go.jp), indicating that only a single copy of *FD1* is conserved. Similar results have been observed in the common bean, chickpea, *M. truncatula* and *L. japonicas* genomes, with genes of the *E1*-like found on chromosomes 9, 1, 2 and 5, respectively (Zhang *et al.* 2016).

The present study demonstrated that *FD1* expression is upregulated under LD conditions in photoperiod sensitive ‘Acc2265’. Other *E1*-like genes, including *E1*, *E1La* and *E1Lb* in soybean and *MtE1L* in *Medicago*, are also more highly expressed under LD than under SD conditions (Jaudal *et al.* 2020, Liu *et al.* 2008, Xu *et al.* 2015). Thus, the LD induced upregulation of expression is consistent among five *E1*-like genes in three legumes. The association of *FD1* expression and flowering response with LD conditions indicates that *FD1* acts as floral repressor in response to LD conditions, similar to findings with the three soybean E1 family genes. In contrast, two independent *MtE1L* knockout mutant lines, in which *MtE1L* was interrupted by a *Tnt1* retrotransposon-tagged insertion, showed both absence of *MtE1L* expression accompanied by late flowering, suggesting that *MtE1L* promotes flowering in *Medicago* (Zhang *et al.* 2016). Although the predicted amino acid sequences of *E1*-like genes are highly homologous, a phylogenic tree indicates that these genes constitute two distinct groups. Group I consisted of eight proteins, FD1, VrE1, PvE1L, E1, E1La, E1Lb, CcE1L1 and CcE1L2, whereas Group II consisted of six proteins, AdE1L, AhE1L, LjE1L, MtE1L, TpE1L and CaE1L. Group I proteins were from SD plants and Group II proteins were from LD plants and day neutral plants, suggesting that the structure of E1 proteins was associated with their functional divergence in photoperiod responsiveness. Further studies are needed to clarify the molecular basis underlying the functional divergence of E1-like proteins.

In the present study, adzuki bean accessions were divided into two *FD1* genotypes, S-type and A-type, using two molecular markers sandwiching the *FD1* coding sequence. Three weedy accessions were classified as A-type and at least two accessions had photoperiod sensitive phenotypes. Small portions of S-type were observed in Shikoku/Kyushu and Chinese regions, suggesting that the S-type photoperiod insensitive genotype arose during the expansion of the area of adzuki bean cultivation on Honshu island. A more extensive sampling of accessions would be required to test this hypothesis.

Among 17 Hokkaido accessions, 15 (88%) were photoperiod insensitive, supporting its impact on adaptation to higher latitudes. However, two A-type accessions, Wasetairyu1 and Akatsuki-dainagon, were photoperiod responsive. In addition, Kita-roman (A-type) exhibited relatively later flowering, independent of day length, than other accessions from Hokkaido. Further study is required to clarify if other flowering pathways, such as thermo-sensitive flowering control, contributes to the adaptation of these three accessions to Hokkaido. Furthermore, there are some exceptions between the genotype of DNA markers and photoperiodic sensitivity, suggesting two possibilities. First, it is necessary to identify the mutations in the *FD1* locus that contribute to photoperiod insensitive. Based on a novel molecular marker being converted from a casual mutation, we will be able to evaluate the association between the genotype at the *FD1* locus and photoperiod sensitivity. Second, it is necessary to determine whether another locus, in addition to *FD1*, contributes to photoperiod sensitivity, as shown in several loci associated with days to first flowering (Isemura *et al.* 2007, Kaga *et al.* 2008, Li *et al.* 2017, Liu *et al.* 2016). The finding of casual mutation(s) at the *FD1* locus and/or another locus associated with photoperiod sensitivity will enhance adzuki bean breeding, improving its latitudinal adaptation by achieving an appropriate flowering time in each target environment, thereby maximizing potential crop yield.

## Author Contribution Statement

KK and YH designed the experiments. YH developed the plant materials. MM analyzed the resequencing data. SM and YT performed phylogenic analysis. YI, SY, YH, TI, TO, MM and KK performed the experiments. KK, YH, MM and YT wrote the first draft. All authors approved the final version of the manuscript.

## Supplementary Material

Supplemental Figure

Supplemental Tables

## Figures and Tables

**Table 1.** The number of light- and circadian-clock related motifs in 3.0 kb of Vigan.02G276800.01 of Shumari and Acc2265

Motif	Shumari	Acc2265
-10PEHVPSBD	5	4
CCAATBOX1	13	12
CIACADIANLELHC	2	2
EBOXBNNAPA	22	22
GATABOX	27	27
GT1CONSENSUS	25	25
GT1CORE	2	2
HDZIP2ATATHB2	1	0
IBOX	1	1
IBOXCORE	9	9
IBOXCORENT	1	1
INRNTPSADB	11	12
REALPHALGLHCB21	9	7
SORLIP1AT	2	2
TATABOX5	11	12
TBOXATGAPB	2	2
