# Supplementary material for: Flowering Date1, a major photoperiod sensitivity gene in adzuki bean, is a soybean floral repressor E1 ortholog
Source: Breed Sci. 2022 Feb 2;72(2):132–40. doi: 10.1270/jsbbs.21051 (PMC9522530; doi:10.1270/jsbbs.21051)
Supplement: Supplementary file 1 — Supplemental Figure [file 72_132_s1.pdf]

Imoto et al./Breeding Science
doi: 10.1270/jsbbs.21051

Supplemental Fig.1 Graphical genotypes and flowering phenotypes of 70 $F_{2:3}$ recombinants between Az02InDel-38274266 and Az02InDel-38970143.

#	F2-ID	Az02InDel-38274266	Az02SNP-38618328	Az02InDel-38621468	Az02InDel-38626644	Az02SNP38628322	Az02SNP-38630660_4	Az02SNP38631002	Az02SNP38631325	Az02InDel-38645437	Az02SNP-38650513	Az02InDel-38650734	Az02gSSR38654173	Az02gInDel38661345	Az02gInDel38668336	Az02gInDel38669973	Az02SSR-38932783	Az02InDel-38943259	Az02SNP-38963646	Az02InDel-38970143	Flowering phneotype		*FDI*
																					F_2	F_3 progeny test	
1	0626F2 ①-2/8-2	H	B	B	B	B	B	B	B	B	B	B	B	B	B	B	B	B	B	B	early	early	B
2	0626F2 ②-3/2-6	H	A	A	A	A	A	A	A	A	A	A	A	A	A	A	A	A	A	A	late	late	A
3	0626F2 ②-3/13-3	H	A	A	A	A	A	A	A	A	A	A	A	A	A	A	A	A	A	A	late	late	A
4	0626F2 ②-4/13-3	H	B	B	B	B	B	B	B	B	B	B	B	B	B	B	B	B	B	B	early	early	B
5	0626F2 ③-1/8-6	H	B	B	B	B	B	B	B	B	B	B	B	B	B	B	B	B	B	B	early	early	B
6	0626F2 ③-2/2-4	H	H	H	H	H	B	B	B	B	B	B	B	B	B	B	B	B	B	B	early	early	B
7	0626F2 ④-2/14-2	A	A	A	A	A	A	A	A	A	A	A	A	A	A	A	H	H	H	H	late	late	A
8	0626F2 ⑤-3/1-7	A	A	A	A	A	A	A	A	A	A	A	A	A	A	A	A	A	A	H	late	late	A
9	0626F2 ⑤-3/8-2	A	H	H	H	H	H	H	H	H	H	H	H	H	H	H	H	H	H	H	late	segregation	H
10	0626F2 ⑤-3/8-4	B	B	B	B	B	B	B	B	B	B	B	H	H	H	H	H	H	H	H	early	early	B
11	0626F2 ⑥-1/6-4	B	H	H	H	H	H	H	H	H	H	H	H	H	H	H	H	H	H	H	late	segregation	H
12	0626F2 ⑥-1/7-1	H	H	H	H	H	H	H	H	H	H	H	H	H	H	H	B	B	B	B	late	segregation	H
13	0626F2 ⑥-3/10-2	H	A	A	A	A	A	A	A	A	A	A	A	A	A	A	A	A	A	A	late	late	A
14	0626F2 ⑦-2/5-7	H	A	A	A	A	A	A	A	A	A	A	A	A	A	A	A	A	A	A	late	late	A
15	0626F2 ⑦-2/16-6	H	B	B	B	B	B	B	B	B	B	B	B	B	B	B	B	B	B	B	early	early	B
16	0626F2 ⑦-4/1-1	A	H	H	H	H	H	H	H	H	H	H	H	H	H	H	H	H	H	H	late	segregation	H
17	0626F2 ⑧-2/7-7	A	H	H	H	H	H	H	H	H	H	H	H	H	H	H	H	H	H	H	late	segregation	H
18	0626F2 ⑧-2/15-1	H	B	B	B	B	B	B	B	B	B	B	B	B	B	B	B	B	B	B	early	early	B
19	0626F2 ⑧-4/1-5	A	A	A	A	A	A	A	A	A	A	A	A	A	H	H	H	H	H	H	late	late	A
20	0626F2 ⑧-4/4-6	H	H	H	H	H	H	H	H	H	H	H	H	H	H	H	A	A	A	A	late	segregation	H
21	0626F2 ⑧-4/6-2	B	B	B	B	B	B	B	B	B	B	B	B	B	B	B	H	H	H	H	early	early	B
22	0626F2 ⑧-4/9-7	H	A	A	A	A	A	A	A	A	A	A	A	A	A	A	A	A	A	A	late	late	A
23	0626F2 ⑨-4/12-4	H	B	B	B	B	B	B	B	B	B	B	B	B	B	B	B	B	B	B	early	early	B
24	0626F2 ⑨-4/12-6	H	A	A	A	A	A	A	A	A	A	A	A	A	A	A	A	A	A	A	late	late	A
25	0626F2 ⑨-4/15-5	A	H	H	H	H	H	H	H	H	H	H	H	H	H	H	H	H	H	H	late	segregation	H
26	0626F2 ⑩-1/7-5	H	A	A	A	A	A	A	A	A	A	A	A	A	A	A	A	A	A	A	late	late	A
27	0626F2 ⑩-1/11-7	H	H	H	H	H	H	H	H	H	H	H	H	H	H	H	B	B	B	B	late	segregation	H
28	0626F2 ⑩-2/3-1	B	B	B	B	B	B	B	B	B	B	B	B	B	B	B	H	H	H	H	early	early	B
29	0626F2 ⑩-2/6-4	H	H	H	H	H	H	H	H	H	H	H	H	H	H	H	H	H	H	B	late	segregation	H
30	0626F2 ⑩-2/9-5	B	B	B	B	B	B	B	B	B	B	B	B	B	B	B	H	H	H	H	early	early	B
31	0626F2 ⑩-2/16-8	H	H	H	H	H	H	H	H	H	H	H	H	H	H	H	H	H	A	A	late	segregation	H
32	0626F2 ⑩-3/15-7	A	H	H	H	H	H	H	H	H	H	H	H	H	H	H	H	H	H	H	late	segregation	H
33	0626F2 11-1/10-8	B	H	H	H	H	H	H	H	H	H	H	H	H	H	H	H	H	H	H	late	segregation	H
34	0626F2 11-2/7-5	B	B	B	B	B	B	B	B	B	B	B	B	B	B	B	B	B	H	H	early	early	B
35	0626F2 11-3/13-7	B	H	H	H	H	H	H	H	H	H	H	H	H	H	H	H	H	H	H	late	segregation	H
36	0626F2 13-2/5-5	H	A	A	A	A	A	A	A	A	A	A	A	A	A	A	A	A	A	A	late	late	A
37	0626F2 13-2/9-7	B	B	B	B	B	B	B	B	B	B	B	B	B	B	B	H	H	H	H	early	early	B
38	0626F2 ①-5/2-2	A	H	H	H	H	H	H	H	H	H	H	H	H	H	H	H	H	H	H	late	segregation	H
39	0626F2 ①-7/7-3	B	B	B	B	B	B	B	B	B	B	B	B	B	B	B	H	H	H	H	early	early	B
40	0626F2 ①-7/9-1	A	H	H	H	H	H	H	H	H	H	H	H	H	H	H	H	H	H	H	late	segregation	H
41	0626F2 ①-7/9-3	H	B	B	B	B	B	B	B	B	B	B	B	B	B	B	B	B	B	B	early	early	B
42	0626F2 ①-7/16-4	B	B	B	B	B	B	B	B	B	B	B	B	B	B	B	H	H	H	H	early	early	B
43	0626F2 ①-8/2-8	H	H	H	H	H	H	H	H	H	H	H	H	H	H	H	B	B	B	B	late	segregation	H
44	0626F2 ①-8/3-3	B	B	B	B	B	B	B	B	B	B	B	B	B	B	B	H	H	H	H	early	early	B
45	0626F2 ①-8/9-7	A	H	H	H	H	H	H	H	H	H	H	H	H	H	H	H	H	H	H	late	segregation	H
46	0626F2 ①-8/13-3	H	B	B	B	B	B	B	B	B	B	B	B	B	B	B	B	B	B	B	early	early	B
47	0626F2 ①-9/2-8	H	A	A	A	A	A	A	A	A	A	A	A	A	A	A	A	A	A	A	late	late	A
48	0626F2 ①-9/6-6	H	A	A	A	A	A	A	A	A	A	A	A	A	A	A	A	A	A	A	late	late	A
49	0626F2 ⑤-5/13-7	H	B	B	B	B	B	B	B	B	B	B	B	B	B	B	B	B	B	B	early	early	B
50	0626F2 ⑤-5/16-2	B	H	H	H	H	H	H	H	H	H	H	H	H	H	H	H	H	H	H	late	segregation	H
51	0626F2 ⑤-6/4-2	H	A	A	A	A	A	A	A	A	A	A	A	A	A	A	A	A	A	A	late	late	A
52	0626F2 ⑤-7/2-6	B	B	B	B	B	B	B	B	B	B	B	B	B	B	B	B	B	B	H	early	early	B
53	0626F2 ⑧-5/3-5	H	B	B	B	B	B	B	B	B	B	B	B	B	B	B	B	B	B	B	early	early	B
54	0626F2 ⑧-6/10-2	H	H	H	H	H	H	H	H	H	H	H	H	H	H	H	H	H	A	A	late	segregation	H
55	0626F2 ⑧-7/1-7	H	A	A	A	A	A	A	A	A	A	A	A	A	A	A	A	A	A	A	late	late	A
56	0626F2 ⑧-7/13-3	H	H	H	H	H	H	H	H	H	H	H	H	H	H	H	B	B	B	B	late	segregation	H
57	0626F2 ⑧-8/8-6	H	A	A	A	A	A	A	A	A	A	A	A	A	A	A	A	A	A	A	late	late	A
58	0626F2 11-5/16-2	H	H	H	H	H	H	H	H	H	H	H	H	H	H	H	A	A	A	A	late	segregation	H
59	0626F2 11-7/10-2	B	B	B	B	B	B	B	B	B	B	B	B	B	B	B	H	H	H	H	early	early	B
60	0626F2 11-7/14-4	B	H	H	H	H	H	H	H	H	H	H	H	H	H	H	H	H	H	H	late	segregation	H
61	0626F2 11-8/10-8	B	B	B	H	H	H	H	H	H	H	H	H	H	H	H	H	H	H	H	late	segregation	H
62	0626F2 12-5/2-4	A	A	A	A	A	A	A	A	A	A	A	A	A	A	A	A	A	A	H	late	late	A
63	0626F2 12-5/4-6	H	H	H	H	H	H	H	H	H	H	H	H	H	H	H	H	H	H	B	late	segregation	H
64	0626F2 12-5/6-4	A	H	H	H	H	H	H	H	H	H	H	H	H	H	H	H	H	H	H	late	segregation	H
65	0626F2 12-5/11-3	A	A	A	A	A	A	A	A	A	A	A	A	A	A	A	H	H	H	H	late	late	A
66	0626F2 12-5/11-7	B	H	H	H	H	H	H	H	H	H	H	H	H	H	H	H	H	H	H	late	segregation	H
67	0626F2 12-6/7-3	A	A	A	A	A	A	A	A	H	H	H	H	H	H	H	H	H	H	H	late	late	A
68	0626F2 15-2/9-7	B	B	B	B	B	B	B	B	B	B	B	B	B	B	B	H	H	H	H	early	early	B
69	0626F2 15-3/5-1	H	A	A	A	A	A	A	A	A	A	A	A	A	A	A	A	A	A	A	late	late	A
70	0626F2 15-3/15-5	B	B	H	H	H	H	H	H	H	H	H	H	H	H	H	H	H	H	H	late	segregation	H

A; Homozygous for the Acc2265 allele

B; Homozygous for the Shumari allele

H; Heterogygous for the Acc2265 allele and the Shumari allele
