# Supplementary material for: Flowering Date1, a major photoperiod sensitivity gene in adzuki bean, is a soybean floral repressor E1 ortholog
Source: Breed Sci. 2022 Feb 2;72(2):132–40. doi: 10.1270/jsbbs.21051 (PMC9522530; doi:10.1270/jsbbs.21051)
Supplement: Supplementary file 2 — Supplemental Tables [file 72_132_s2.pdf]

Supplemental Table 1 Characteristic of the insertion and deletion, microsatellite, CAPS and dCAPS markers developed in the present study.

Position[a] (bp)	Marker name	Type	Primer sequence (5'-->3') Forward	Primer sequence (5'-->3') Reverse	PCR product size (bp) Shumari	PCR product size (bp) Acc2265	Restriction enzyme	Restriction fragment length (bp) Shumari	Restriction fragment length (bp) Acc2265
38,118,117	Az02InDel-38118117	InDel	CAAGAGCAGAAGCTCCCATT	TCCTCAAATTCATACCATTGAA	186	194			
38,261,085	Az02SNP-38261085	CAPS	AGCCTTCGTTGCATTTCATT	CCCTTCACATTCATGAGACCT	183	183	*Dpn* I	183	78 + 105
38,274,266	Az02InDel-38274266	InDel	GACATCACCAGAGGCACTGA	TGTGACCCACGGATGACTTA	208	194			
38,535,655	Az02SNP-38535655	CAPS	CCAATCGGCTAACAAAGCAT	TTTCCATTGTATTACACGAGTTTC	238	238	*Taq* I	138+75+25	163+75
38,617,757	Az02SNP-38617757	dCAPS	AAAAATAACCAAAACTAATAATTTTGTTC	CCTTCAGTCCCACCTTTCAA	110	110	*Mae* I	81+29	110
38,618,328	Az02SNP-38618328	dCAPS	TAAGTCATTAAATGATGTAAATCCAGAAG	TCAGCTTGATACACAAAGGACAA	244	244	*Mbo* II	215+29	244
38,621,468	Az02InDel-38621468	InDel	CAGGTTTCACATGTTTAGTGTGG	GGTGGTTTCGAGCTTTCTTG	216	207			
38,626,644	Az02InDel-38626644	InDel	TGACCAAACTTTCTCACGACA	CATGGCATGTCTAACCTCATT	166	178			
38,627,816	Az02InDel-38627816	InDel	CATACCATATTCAAAGGAGCCTA	TGTCAAATGTCCATATCCTTGTG	230	270			
38,628,322	Az02SNP-38628322	CAPS	ACACCCCTAATTGCCACAAG	TTGTTGGTCCACTCACATTCA	188	188	*Dde* I	188	137+51
38,630,660	Az02SNP-38630660_4	CAPS	GCTCCTTTTCATCTCCAGGGT	AGCCTCACTTCCATTTCACAAAT	173	173	*Taq* I	138+35	173
38,631,002	Az02SNP-38631002	dCAPS	TTCTCTTGCTTTCTTTTTTAGTTTCCATG	TACGCCAAAGCAGAGATCAAT	180	180	*Mbo* I	134+29+17	163+17
38,631,325	Az02SNP-38631325	dCAPS	ACTTGCAATCAGAATCTAAATACTTTCTC	TGCCTTTTTGTCATCGGAAT	103	103	*Dde* I	103	74+29
38,645,437	Az02InDel-38645437	InDel	TAAGAGTTTATGAGATCCAAAT	AAAAGGATAGAATTTTGAATGAA	152	184			
38,650,513	Az02SNP-38650513	dCAPS	CGTTTTAACCTAAACAAAGTAATAC	GCGTGGGAAAGAAAAATTCA	228	228	*Bsr* I	203+25	228
38,650,734	Az02InDel-38650734	InDel	TTTCCCACGCAAAAATATAA	AACACTTAGCCACTTGTGAGA	150	171			
38,654,173	Az02SSR-38654173	SSR	CTCCACAGACACAACCCCTT	TCGTTGCGTGCTTCTTTTCC	193	185			
38,661,345	Az02InDel-38661345	InDel	AGTGGGAGGAACGAGGGTAA	CATTGACCCAGATTGCGTGC	103	113			
38,668,336	Az02InDel-38668336	InDel	GACCGGATCATCTATGCCCG	ACAAAACAACCACGACTGCG	141	118			
38,669,790	Az02InDel-38669790	InDel	TGGAGTTGGATAAAAGGGGCT	TCAATAACTGCACTGAGACAGA	114	110			
38,669,973	Az02InDel-38669973	InDel	TGGATCTTGGAGTTGGAGCA	TCCAAGAGGTCATTTGGTGAA	139	150			
38,872,702	Az02InDel-38872702	InDel	TCGAGTATCGGGTGGTGTTC	TGGACACCTTTTGCAGCTCA	114	164			
38,881,961	Az02InDel-38881961	InDel	TCAAAGTGTCCTTTAGGTCAAACA	TCATGATGTTGTCCACTTGATCT	215	237			
38,891,141	Az02InDel-38891141	InDel	CAAGCAGAGGTTCGATGTCA	ATTGACCACCACCAGAGTCC	222	242			
38,916,233	Az02InDel-38916233	InDel	TTAGTGGTCCCAATCCCAAA	GCCGAATCCAAAAATTTCCT	240	221			
38,932,783	Az02SSR-38932783	SSR	AAAATAAAAGCAGAGGGGAAA	CTGTTGAAATTCACGCCATT	330	290			
38,933,834	Az02InDel-38933834	InDel	TGCAAGACTCCAAACTTTCTC	TTTTACTCTTAGGAAGGGATTTGAA	200	188			
38,943,259	Az02InDel-38943259	InDel	TGAGATGGTTCAAACTATCAACG	TGACAACCTAATTATGACCCAAT	159	169			
38,963,646	Az02SNP-38963646	dCAPS	AGTTACCAATAGAATGGTTTAGGAGCTGA	CGTAACTGGCTTCCGATTCT	123	123	*Dde* I	94+29	123
38,970,143	Az02InDel-38970143	InDel	TCAGTTGTTGTTGATGAAGTGG	TTTTGTCGAAACGCGTGTAA	140	130			
38,974,158	Az02InDel-38974158	InDel	AATGTCCAAGCCAATTCCGC	ATGTGAGGGTAGGAGGGTCC	115	152			

a; Physical position of polymorphic position (bp) on chromosome 2 avaiable at Vigna Genome Server (http://viggs.dna.affrc.go.jp).

Supplemental Table 2 Adzuki bean accessions used in the present study. Genotypes of two molecular markers close to FD1 and days from sowing to initial flowering under short day (SD) and long day (LD) were characterized in the present study.

Accession	Cultivar/Accession Name	Status (Cultivar registration)	Region, prefecture /Country	Genotype*		Days to flowering	
				Az02SNP-38631265	Az02InDel-38627816	SD	LD
Acc2773	Nagano landrace94	Landrace	Nagano/Japan	A	A	29.3	61.0
Acc2774	Ibaraki landrace4	Landrace	Ibaraki/Japan	A	A	-	-
Acc2775	Tokushima landrace19	Landrace	Tokushima/Japan	A	A	-	-
Acc2776	Iwate landrace239	Landrace	Iwate/Japan	S	S	34.0	46.3
Acc2777	Iwate landrace240	Landrace	Iwate/Japan	S	S	29.7	61.0
Acc2778	Iwate landrace241	Landrace	Iwate/Japan	S	S	-	-
Acc2779	Saitama landrace14	Landrace	Saitama/Japan	S	S	29.7	30.3
Acc2780	Ibaraki landrace5	Landrace	Ibaraki/Japan	S	S	29.7	32.0
Acc2781	Fukusima landrace106	Landrace	Fukushima/Japan	S	S	-	-
Acc2782	Iwate landrace242	Landrace	Iwate/Japan	S	S	29.0	56.0
Acc2783	Iwate landrace243	Landrace	Iwate/Japan	S	S	-	-
Acc2784	Akita landrace104	Landrace	Akita/Japan	S	S	-	-
Acc2786	Iwate landrace244	Landrace	Iwate/Japan	A	A	36.3	54.3
Acc2787	Iwate landrace245	Landrace	Iwate/Japan	S	S	-	-
Acc2788	China134	Landrace	China	A	A	29.3	61.0
Acc2789	China135	Landrace	China	A	A	35.0	59.3
Acc2790	China136	Landrace	China	A	A	29.0	53.0
Acc2791	China137	Landrace	China	A	A	-	-
Acc2792	China138	Landrace	China	A	A	35.7	32.0
Acc2793	China139	Landrace	China	A	A	27.7	61.0
Acc2794	China140	Landrace	China	A	A	34.7	61.0
Acc2795	China141	Landrace	China	A	A	32.3	26.7
Acc2796	China142	Landrace	China	A	A	33.0	35.7
Acc2797	China143	Landrace	China	A	A	45.0	57.3
Acc2798	China144	Landrace	China	A	A	34.7	60.0
Acc2799	China145	Landrace	China	A	A	31.3	61.0
Acc2800	Wakayama landrace26	Landrace	Wakayama/Japan	A	A	27.7	61.0
Acc2801	Iwate landrace246	Landrace	Iwate/Japan	S	S	-	-
Acc2802	Unknown (Tohoku)	Landrace	Touhoku region/Japan	A	A	-	-
Acc2803	Iwate landrace247	Landrace	Iwate/Japan	S	S	-	-
Acc2804	Akita landrace105	Landrace	Akita/Japan	S	S	-	-
Acc2805	Aomori landrace97	Landrace	Aomori/Japan	S	S	-	-
Acc2806	Aomori landrace98	Landrace	Aomori/Japan	S	S	30.7	61.0
Acc2807	Aomori landrace99	Landrace	Aomori/Japan	S	S	-	-
Acc2808	Aomori landrace100	Landrace	Aomori/Japan	S	S	-	-
Acc2809	Aomori landrace101	Landrace	Aomori/Japan	S	S	-	-
Acc2810	Aomori landrace102	Landrace	Aomori/Japan	S	S	-	-
Acc2811	China146	Landrace	China	A	A	39.0	61.0
Acc2812	Iwate landrace248	Landrace	Iwate/Japan	S	S	-	-
Acc2813	Iwate landrace249	Landrace	Iwate/Japan	S	S	-	-
Acc2814	Iwate landrace250	Landrace	Iwate/Japan	S	S	-	-
Acc2815	Iwate landrace251	Landrace	Iwate/Japan	A	A	-	-
Acc2816	Iwate landrace252	Landrace	Iwate/Japan	S	S	-	-
Acc2817	China147	Landrace	China	A	A	31.7	28.3
Acc2818	China148	Landrace	China	A	A	34.7	61.0
Acc2819	China149	Landrace	China	A	A	38.0	31.7
Acc2820	China150	Landrace	China	A	A	44.7	61.0
Acc2821	China151	Landrace	China	A	A	28.0	61.0
Acc2822	China152	Landrace	China	A	A	32.0	58.0
Acc2823	China153	Landrace	China	A	A	31.7	61.0
Acc2824	Okinawa landrace32	Landrace	Okinawa/Japan	A	A	29.3	50.7
Acc2825	Iwate landrace253	Landrace	Iwate/Japan	A	A	28.3	41.7
Acc2826	Iwate landrace254	Landrace	Iwate/Japan	S	S	-	-
Acc2827	China154	Landrace	China	A	A	30.3	61.0
Acc2828	Kochi landrace30	Landrace	Kochi/Japan	S	S	41.7	37.3
Acc2862	Nagano landrace95	Landrace	Nagano/Japan	S	S	31.3	58.0
Acc2863	Tokushima landrace20	Landrace	Tokushima/Japan	A	A		
Acc2864	Tokushima landrace21	Landrace	Tokushima/Japan	A	A	30.7	52.3
Acc2865	Unknouwn (Japan) 5	Landrace	Japan	S	S	-	-
Acc2866	Fukushima landrace108	Landrace	Fukushima/Japan	A	A	32.3	61.0
Acc2867	Ibaraki landrace6	Landrace	Ibaraki/Japan	A	A	31.0	61.0
Acc2868	Ibaraki landrace7	Landrace	Ibaraki/Japan	A	A	32.3	61.0
Acc2869	Akita landrace106	Landrace	Akita/Japan	S	S	-	-
Acc2870	Akita landrace107	Landrace	Akita/Japan	A	A	-	-
Acc2871	Tokushima landrace22	Landrace	Tokushima/Japan	A	A	-	-
Acc2872	Kochi landrace31	Landrace	Kochi/Japan	A	A	-	-
Acc2873	Kochi landrace32	Landrace	Kochi/Japan	A	A	31.7	61.0
Acc2874	Kochi landrace33	Landrace	Kochi/Japan	A	A	-	-
Acc2875	Aomori landrace109	Landrace	Aomori/Japan	A	A	28.3	61.0
Acc2876	China155	Landrace	China	S	S	31.7	34.0
Acc2877	China156	Landrace	China	A	A	27.3	61.0
Acc2878	Shimane landrace110	Landrace	Shimane/Japan	A	A	33.3	61.0
Acc2879	Okinawa Landrace33	Landrace	Okinawa/Japan	A	A	-	-
Acc2880	Hyogo landrace113	Landrace	Hyogo/Japan	A	A	-	-
Acc2881	Chiba Noadzuki 4	Weedy	Chiba/Japan	A	A	-	-
Acc2882	Akita landrace108	Landrace	Akita/Japan	S	S	-	-
Acc2883	Akitalandrace109	Landrace	Akita/Japan	S	S	28.7	61.0
Acc2884	Akita landrace110	Landrace	Akita/Japan	A	A	-	-
Acc2885	Kochi landrace34	Landrace	Kochi/Japan	A	A	-	-
Acc2886	Kochi landrace35	Landrace	Kochi/Japan	A	A	31.7	32.3
Acc2887	Kochi landrace36	Landrace	Kochi/Japan	A	A	-	-
Acc2888	Kochi landrace37	Landrace	Kochi/Japan	A	A	-	-
Acc2889	Kochi landrace38	Landrace	Kochi/Japan	A	A	-	-
Acc2890	Kochi landrace39	Landrace	Kochi/Japan	A	A	-	-
Acc2891	Kochi landrace40	Landrace	Kochi/Japan	A	A	31.7	35.0
Acc2892	Iwate landrace273	Landrace	Iwate/Japan	S	S	-	-
Acc2893/JP90790	CED97021-4	Weedy	Yamagata/Japan	A	A	34.3	61.0
Acc2894/JP90811	CED97069-12	Weedy	Hyogo/Japan	A	A	32.3	61.0
Acc2895	Iwate landrace274	Landrace	Iwate/Japan	S	S	29.7	61.0
Acc2896	Ehime landrace16	Landrace	Ehime/Japan	A	A	30.0	61.0
Acc2898	Tokushima landrace23	Landrace	Tokushima/Japan	A	A	-	-
Acc2899	Tokushima landrace24	Landrace	Tokushima/Japan	A	A	30.0	51.3
Acc2900	Tokushima landrace25	Landrace	Tokushima/Japan	A	A	-	-
Acc2901	Tokushima landrace26	Landrace	Tokushima/Japan	A	A	32.7	34.3
Acc2902/JP31219	COL/TOKUSHIMA/1967	Landrace	Tokushima/Japan	A	A	30.7	45.3
Hinpo1	Chagarawase	Landrace (1914)	Hokkaido/Japan	S	S	34.0	35.3
Hinpo6	Wasetairyu1	Pure line breeding (1930)	Hokkaido/Japan	A	A	31.0	53.3
Hinpo9	Takara-shozu	Cross breeding (1959)	Hokkaido/Japan	S	S	36.7	30.7
Hinpo10	Kotobuki-shozu	Cross breeding (1971)	Hokkaido/Japan	S	S	33.7	38.0
Hinpo11	Hikari-shozu	Cross breeding (1964)	Hokkaido/Japan	S	S	37.0	34.3
Hinpo12	Sakae-shozu	Cross breeding (1973)	Hokkaido/Japan	S	S	37.3	34.3
Hinpo13	Akatsuki-dainagon	Cross breeding (1976)	Hokkaido/Japan	A	A	40.0	57.3
Hinpo32	Ken-3	Pure line breeding (1927)	Hokkaido/Japan	A	A	-	-
Hinpo140	Tsuru-shozu (Sarabetsu)	Landrace (1962)	Hokkaido/Japan	S	S	37.3	36.0
Hinpo273	Hayate-shozu	Cross breeding (1976)	Hokkaido/Japan	A	A	37.7	33.0
Hinpo274	Erimo-shozu	Cross breeding (1981)	Hokkaido/Japan	S	S	39.0	33.7
Hinpo275	Akane-dainagon	Cross breeding (1974)	Hokkaido/Japan	A	A	-	-
Hinpo277	Hatsune-shozu	Cross breeding (1985)	Hokkaido/Japan	A	A	38.0	34.0
Hinpo278	Beni-dainagon	Cross breeding (1986)	Hokkaido/Japan	A	A	-	-
Hinpo280	Kensaki (Kari No. 6)	Landrace (1905)	Hokkaido/Japan	A	A	35.0	37.0
Hinpo283	Chunagon (Kari No. 47)	not avaiable	Iwate/Japan	A	A	-	-
Hinpo293	Tochigi Maruba No. 1 (Kari No. 79)	not avaiable	Iwate/Japan	A	A	-	-
Hinpo297	Takahashi-wase (Kari No. 87)	Cross breeding (1924)	Iwate/Japan	A	A	31.7	32.0
Hinpo312	Tsurushozu (Kari No. 138)	not avaiable	Iwate/Japan	S	S	-	-
Hinpo384	Noto-shozu	Landrace	Ishikawa/Japan	A	A	33.3	28.7
Hinpo399	Urasa	Landrace	Shimane/Japan	S	S	-	-
Hinpo400	Kuro-shozu	Landrace	Okayama/Japan	A	A	28.7	61.0
Ikukei10002	Sahoro-shozu	Cross breeding (1989)	Hokkaido/Japan	A	A	-	-
Ikukei10003	Kamui-dainagon	Cross breeding (1990)	Hokkaido/Japan	A	A	-	-
Ikukei10005	Akeno-wase	Cross breeding (1992)	Hokkaido/Japan	A	A	-	-
Ikukei10008	Kita-no-otome	Cross breeding (1994)	Hokkaido/Japan	S	S	39.0	36.7
Ikukei10011	Toiku No.130	Breeding line (1990)	Hokkaido/Japan	S	S	-	-
Ikukei10014	Hokuto-dainagon	Cross breeding (1996)	Hokkaido/Japan	A	A	-	-
Ikukei10021	Shumari	Cross breeding (2000)	Hokkaido/Japan	S	S	32.8	32.1
Ikukei10024	Toyomi-dainagon	Cross breeding (2001)	Hokkaido/Japan	A	A	-	-
Ikukei10027	Kita-hotaru	Cross breeding (2004)	Hokkaido/Japan	S	S	36.5	34.3
Ikukei10028	Kita-roman	Cross breeding (2005)	Hokkaido/Japan	A	A	42.0	48.7
Ikukei20006	Tokei No.486	Breeding line (1990)	Hokkaido/Japan	S	S	-	-
Acc2265		Landrace	Bhutan	A	A	36.9	61.0

*: A and S represent Acc2265 type and Shumari type, respectively.

Supplemental Table 3 Distribution of flowering date in the F_2 population of a cross between Shumari and Acc2265. Plant materials were cultivated at the experimental field of TAES in 2016.

25-Jul	27-Jul	29-Jul	1-Aug	3-Aug	5-Aug	8-Aug	10-Aug	12-Aug	13-Aug ~	Total
0	7	44	212	50	38	7	3	2	788	1,151
